# Exploration in the Mechanism of Zhisou San for the Treatment of Cough Variant Asthma Based on Network Pharmacology

**DOI:** 10.1155/2022/1698571

**Published:** 2022-06-29

**Authors:** De-hai Guo, Jin-ping Hao, Xing-jie Li, Qing Miao, Qiong Zhang

**Affiliations:** ^1^Xiyuan Hospital, China Academy of Chinese Medical Sciences, Beijing 100091, China; ^2^Department of Pharmacy, Xuanwu Hospital of Capital Medical University, Beijing 100053, China; ^3^Research Center for Clinical and Translational Medicine, Fifth Medical Center, General Hospital of Chinese PLA, Beijing 100039, China

## Abstract

**Background:**

Cough variant asthma (CVA) has no definitive diagnosis or pathogenic causes, and there is currently no effective and safe treatment.

**Methods:**

The network pharmacology was employed to investigate possible targets of Zhisou San (ZSS) in CVA treatment. The main chemical constituents of seven herbs in ZSS were collected based on the TCMSP. To explain the main mechanism, we sequentially screened the targets of each active ingredient and constructed the network of “herb-ingredient-target-disease.” The core targets of ZSS were further confirmed by the molecular docking analysis. Furthermore, pulmonary function, histopathology, and biochemical assays in mice were used to investigate the effect of ZSS on the treatment of CVA.

**Results:**

A total of 137 active ingredients and 86 potential targets for the ZSS in the treatment of CVA were screened, which were connected with the regulation of inflammatory response and immune balance, such as IL-17 signaling pathway, Th17 cell differentiation, TNF signaling pathway, Toll-like receptor signaling pathway, MAPK signaling pathway, T-cell receptor signaling pathway, Th1 and Th2 cell differentiation, and other signaling pathways closely related to the pathogenesis of CVA. Thereinto, 29 core targets contained 8 of the highest scores and could evidently bind to components such as stigmasterol, quercetin, stemoninine B, luteolin, and *β*-sitosterol predicted by molecular docking. Furthermore, experiments in vivo were conducted for further validation that ZSS had essential effects on lung function and histopathology as well as the inflammatory state in CVA mice, which was significantly related to regulating the Th17/Treg immune balance to reduce inflammation as the important pharmacological mechanism.

**Conclusion:**

This study revealed that ZSS has multicomponent and multipathway characteristics of ZSS in the treatment of CVA, which was primarily associated with inflammation and Th17/Treg immune balance. This study provides a scientific foundation for systematically elaborating the pharmacological activities and mechanism of ZSS, as well as explaining the reliability of the TCM compatibility theory.

## 1. Introduction

Cough variant asthma (CVA) is a type of asthma in which the chronic and recurrent cough is the main or sole clinical manifestation [1]. It is prone to clinical misdiagnosis and missed diagnosis as patients have no wheezing, chest tightness, dyspnea, and other symptoms [2]. CVA occupies the first place among many causes of chronic cough in adults, which usually occurs at night or in the morning [3]. If CVA symptoms are unable to be effectively controlled, 30–40% of CVA patients will develop typical asthma [4, 5]. All of the aforementioned factors not only reduce patients' quality of life but also increase the financial burden. However, the pathogenesis of CVA is still unclear, and currently, theories mainly include chronic airway inflammation, airway hyper-responsiveness, cough receptor sensitivity, and airway remodel ligand [6]. At present, western medical treatments for CVA are antibiotics, antihistamines, glucocorticoids, acid suppressants, gastric motility drugs, expectorants, antitussives, etc. [5]. Although there is a certain effect, most medications have evident adverse effects and withdrawal symptoms that coincide with a recurrence of illness [7]. The clinical practice showed that traditional Chinese medicine (TCM) has obvious efficacy in CVA treatment characterized by the wholism and dialectic to ensure the safety, effectiveness, and less toxic and side effects [8, 9]. Therefore, it is of great practical significance to explore the therapy of TCM in treating CVA.

TCM formula mainly refers to two or more herbs used in combination, as the primary form of clinical application in TCM, which meets the demand for treatment of multifactor diseases requiring interactions with multitarget in modern medicine. Zhisou San (ZSS), a famous prescription of Dr. Cheng Guopeng in the Qing dynasty of China, composed of seven herbs including *Platycodon grandiflorum* (Jacq.) A. DC. (JG), *Nepeta cataria* L. (JJ), *Aster tataricus*L.f. (ZW), *Stemonasessilifolia* (Miq.) Miq. (BB), *Cynanchumstauntonii* (Decne.) Schltr. ex H.Lév. (BQ), *Glycyrrhiza uralensis* Fisch. (GC), and *Citrus reticulata* Blanco (CP) has been used in relieving cough, eliminating phlegm, and diffusing the lung for hundreds of years [10]. This formulation is the favored method to treat chronic cough in TCM owing to its rigorous compatibility with the property of mild and warm rather than hot or cool, and it has shown promising effects in the treatment of CVA [11, 12]. Nevertheless, due to the complexity of components, it is difficult to clarify the specific roles of effective substances in CVA, and the scientific basis to reasonably explain the pharmacodynamic mechanism of this prescription from the whole to the molecular level also needs to be further probed.

Recently, network pharmacology, whose theme is “network target,” has provided efficient means and new ideas for the systematic study of the pharmacodynamics mechanism of TCM with special features such as multitarget and multichannel [13, 14]. Network pharmacology integrates bioinformatics and systems biology to explore the interaction relation of “ingredients-targets-pathways,” which reveal the intricate biological network linking drugs, genes, targets, and diseases to analyze and predict the pharmacological mechanism of drugs [15]. Given that TCM has characteristics of multicomponent, multipathway, multitarget, and synergistic action focusing on the whole rather than the locality, network pharmacology corresponds highly with the TCM theory in terms of integrity and systematicness, which also provides a new opportunity for the in-depth study of TCM prescription in predicting targets, screening the material basis for clinical application, and providing insights into the underlying mechanisms of TCM.

In the present study, a network pharmacology approach, which included target prediction, network analysis, and pathway enrichment, was employed to predict bioactive compounds and therapeutic targets of ZSS on CVA. Then, molecular docking was performed to validate major targets by computer modeling technology for component-target binding affinities. Finally, using the CVA mouse model, we conducted in vivo validation by pulmonary functional, histopathology, and biochemical assays. This study presented a systematical approach for the exploration of potential molecular mechanisms in the CVA treatment by ZSS, which was also expected to guide the experimental study and the clinical application of ZSS, as well as to provide a reference for the network pharmacology research of other TCM prescription.

## 2. Materials and Methods

### 2.1. Screening of the Potentially Active Compounds and the Related Targets in Zhisou San

All chemical compounds of 7 herbs from Zhisou San (ZSS) were searched through the Traditional Chinese Medicine Systems Pharmacology database and analysis platform (TCMSP, https://old.tcmsp-e.com/tcmsp.php), and they were screened based on two key parameters of absorption, distribution, metabolism, and excretion (ADME) such as oral bioavailability (OB) ≥30% and drug-likeness (DL) ≥0.18 for preliminary screening to obtain the active compounds and their targets. In addition, targets of several unpredicted active compounds were added based on available literature. After the screening, compound-related targets were verified using the Uniprot Protein Database (https://www.uniprot.org) and converted to the corresponding gene name in order to standardize the protein target information.

### 2.2. Collection of Related Targets of Cough Variant Asthma

Taking “cough variant asthma” as a keyword, the potential targets of CVA were explored in the databases including GeneCards (https://www.genecards.org), OMIM (http://www.omim.org), DRUGBANK (https://www. drugbank.ca), and DisGeNET (https://www.disgenet.org). In the GeneCards database, a higher score indicates that the gene is closely associated with developmental disorders. If there were too many targets associated with disease, targets with scores greater than the median would be set as potential targets that were then merged and removed duplicates to construct the CVA-related targets set.

### 2.3. Construction of Compound-Target Protein Interaction Network of Zhisou San in Treating Cough Variant Asthma

In order to clarify the interaction of relevant targets of ZSS in the treatment of CVA, the intersection between compound-related targets of ZSS and CVA-related targets was firstly taken by R language to draw the Venn diagram. Then, intersection targets were imported into the STRING 11.0 database (https://string-db.org) to construct a (protein-protein interaction) PPI network, in which the species were set as “Homo sapiens,” minimum interaction threshold was set as “highest confidence” (>0.9), and the rest of the parameters were on their default settings. The PPI network is classed as an undirected graph since the interactions between proteins are reciprocal. Some modules with high density in the PPI complex network are considered the biologically significant collections, which can be obtained through further analysis of the PPI network by the MCOD plug-in of Cytoscape 3.7.2. Core targets were selected from the module with the highest score to establish the relationship network of ZSS in the treatment of CVA.

### 2.4. Gene Ontology and Kyoto Encyclopedia of Genes and Genomes Pathway Enrichment Analysis

Metascape platform (https://metascape.org/gp/index.html) has comprehensive annotation capabilities and monthly updates the data of gene annotation. Targets of ZSS in the CVA treatment were input into the Metascape platform under the restricted species as humans to perform the Gene Ontology (GO) and Kyoto Encyclopedia of Genes and Genomes (KEGG) pathways enrichment analysis. GO enrichment was mainly reflected in three aspects: molecular function (MF), biological process (BP), and cellular components (CC). The result set was sorted by the value of -log(P), and the top biological processes or pathways were screened out for further analysis.

### 2.5. Molecular Docking Prediction

Molecular docking is a simulation method that studies the interactions between molecules and predicts binding mode and affinity, which has become an important skill in the field of computer-assisted drug research. AutoDock 4.0 software was used for molecular docking. The top core targets of ZSS in the treatment of CVA were selected according to the value of the MCOLD score, and their crystal structures were downloaded from the protein database (https://www1.rcsb.org/). Next, these crystal structures were modified with the software of PyMol to remove the original ligand and water molecules and saved in the PDBQT format. TCMSP database (https://old.tcmsp-e.com/tcmsp.php) was used to collect and download the two-dimensional structures of related compounds as small-molecule ligands and save them in the MOL2 format. Finally, 3D crystal structures of protein targets and the corresponding compounds ligand were imported into AutoDock 4.0 software for molecular docking.

### 2.6. Herbal Materials and Mice Treatment

Zhisou San (ZSS) is composed of seven herbs including 10 g Platycodonis Radix (*Platycodon grandiflorum* (Jacq.) A. DC.) (JG), 10 g Schizonepetae Herba (*Nepeta cataria* L.) (JJ), 15 g Asteris Radix et Rhizoma (*Aster tataricus*L.f.) (ZW), 10 g Stemonae Radix (*Stemona sessilifolia* (Miq.) Miq.) (BB), 15 g Cynanchi Stauntonii Rhizoma et Radix (*Cynanchumstauntonii* (Decne.) Schltr. ex H.Lév.) (BQ), 10 g Glycyrrhizae Radix et Rhizoma (*Glycyrrhiza uralensis* Fisch.) (GC), and 15 g Citri Reticulatae Pericarpium (*Citrus reticulata* Blanco) (CP), which were added to boiling water about 10 times the volume of herbs, extracted twice and 30 mins each time. Then, the extract was filtrated and evaporated by a water bath and lyophilized for containing 6.25 g of raw herbs per gram, kept in 4°C cold storage. The dry powder was dissolved in the saline used in this experiment.

Male BALB/c mice at 2-3 months of age were purchased from Beijing SiPeiFu Biotechnology Co., Ltd. (Beijing, China) and kept on a 12 h light-dark cycle with free access to food and water. After one week of acclimatization, mice were first randomly divided into 4 groups (*n* = 6 per group): control group (Ctrl), model group (Mod), low dose of ZSS group (LZSS), and high dose of ZSS group (HZSS). On days 1, 7, and 14, mice in the control group were intraperitoneally injected with 200 *μ*l normal saline, and mice in other groups were intraperitoneally injected with 200 *μ*l sensitization solution (100 *μ*g ovalbumin and 20 *μ*g aluminum hydroxide). One week after the last injection, all groups except the Ctrl were given 0.5% ovalbumin for ultrasonic atomization 30 min a day; then, all mice were, respectively, treated with saline, ZSS extracts at the dose of 0.75 g/kg and 1.5 g/kg once per day for 14 consecutive days. All experiments were repeated three times at least and approved by the Animal Care and Ethics Committee at the Fifth Medical Centre, Chinese PLA (People's Liberation Army).

### 2.7. Pulmonary Function and Histological Analyses

The airway reactivity of mice was measured by a noninvasive pulmonary function instrument (Buxco, USA). Mice were placed in a flow-type global plethysmography box, and one mouse was monitored in each round. According to the set procedure, the basal value, the value of normal saline, and the value of excitation by 3.12 mg/ml. 6.25 mg/ml, 12.5 mg/ml, 25 mg/ml, and 50 mg/ml methacholine (Mch) were measured successively to calculate the enhanced pause (Penh) value of each Mch concentration through the system. The volume of each Mch dose was 0.1 ml, and the atomizing time was 1 min. The left lung was fixed with a 10% formalin neutral buffer solution, embedded in paraffin, sectioned to a thickness of 5 *μ*m, and stained with hematoxylin and eosin (HE).

### 2.8. Cytokine ELISA

Serum levels of IL-1*β*, IL-6, TNF-a, IL-17, and IL-10 in mice were measured with ELISA kits according to the manufacturer's instructions. All determinations were performed in triplicate.

### 2.9. Flow Cytometry

The spleen was isolated and meshed through 45 *μ*m cell strainers (Corning BD Falcon) in PBS to obtain single-cell suspensions. Spleen cells were stained with CD3-FITC (BD) and CD4-percp-cy5.5 (BD), as well as CD4-FITC (BD) and CD25-PE (BD). After the primary staining, spleen cells were intracellularly stained with IL-17A-PE (eBioscience) and Foxp3-PE (eBioscience) in Perm/Wash for 40 min at room temperature avoiding light. Cells were then washed with Perm/Wash, resuspended in PBS containing 1 *μ*g/ml DAPI. Spleen immunostaining was recorded by LSRII/Fortessa (BD Biosciences), and data were analyzed using FlowJo software V10.

### 2.10. Statistics

All results were presented as means ± standard deviations (SD). Statistical analyses were analyzed using the SPSS 13.0 package (SPSS Inc., Chicago, IL, USA) by analysis of variance (ANOVA). Neuman–Keuls or Tukey's multiple-comparisons test was conducted as ANOVA justified post hoc comparisons between group means. *P* < 0.05 was considered to be significant.

## 3. Results

### 3.1. Identification of Potentially Active Compounds and Related Targets in Zhisou San

After screening from the TCMSP database with two key parameters of (OB) ≥30% and drug-likeness (DL) ≥0.18, we basically captured the information that 4 compounds and 92 targets were from JG, 10 compounds and 307 targets were from JJ, 13 compounds and 381 targets were from ZW, 28 compounds and 288 targets were from BB, 4 compounds and 47 targets were from BQ, 86 compounds and 1752 targets were from GC, and 5 compounds and 95 targets were from CP. Removing duplicates, a total of 137 compounds and 276 related targets were collected from the prescription of ZSS (Table S1).

### 3.2. Prediction of Targets in the CVA Treatment by Zhisou San and PPI Network Analysis

**Figure 1:**
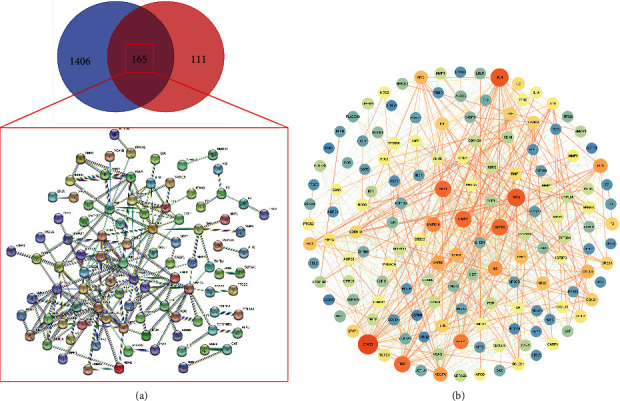
All targets of Zhisou San (ZSS) and cough variant asthma (CVA) were identified and a PPI network was drawn with the overlapped targets of ZSS in treating CVA.

1052 CVA-related targets were obtained from the GeneCards database, and other relevant targets were supplemented combined with the OMIM, Disgenet, and DRUGBANK databases. Removing duplicates after merging, a total of 1571 CVA-related targets were finally collected. We then obtained the 165 intersected targets between the compounds of ZSS and CVA as the potential therapeutic targets (Figure 1(a)).

**Figure 2:**
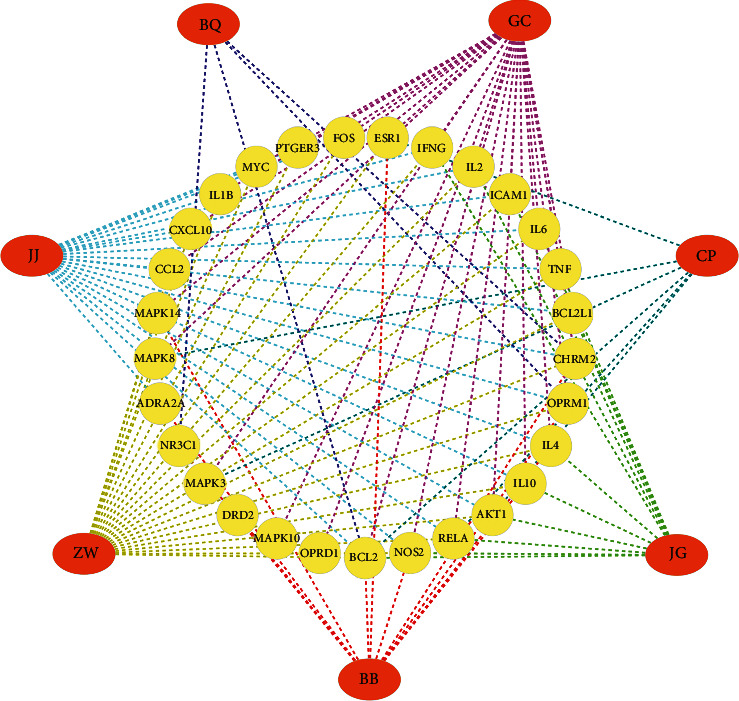
165 intersecting targets of Zhisou San (ZSS) in treating cough variant asthma (CVA) were imported into the STRING data platform and 29 core targets attributing to the herbs of ZSS were selected out from the module with high scores calculated by MCOD plug-in of Cytoscape software.

These 165 relevant targets were imported into the STRING data platform to obtain the PPI network in the CVA treatment by ZSS (Figure 1(b)). 29 core targets were selected out from the module with high scores calculated by the MCOD plug-in of Cytoscape 3.7.2 (Figure 2), and the associated active compounds were shown in Table S2, among which 8 targets with the highest score of 7 including *β*2-adrenergic receptor (*β*2-ADR), delta-type opioid receptor (OPRD1), C-X-C motif chemokine 10 (CXCL10), muscarinic acetylcholine receptor M2 (CHRM2), D(1) dopamine receptor (DRD1), prostaglandin E2 receptor EP3 subtype (PTGER3), mu-type opioid receptor (OPRM1), and intercellular adhesion molecule 1 (ICAM1) are listed in Table 1. Consequently, these 8 targets were selected for molecular docking verification.

### 3.3. GO and KEGG Pathway Enrichment Analyses

**Figure 3:**
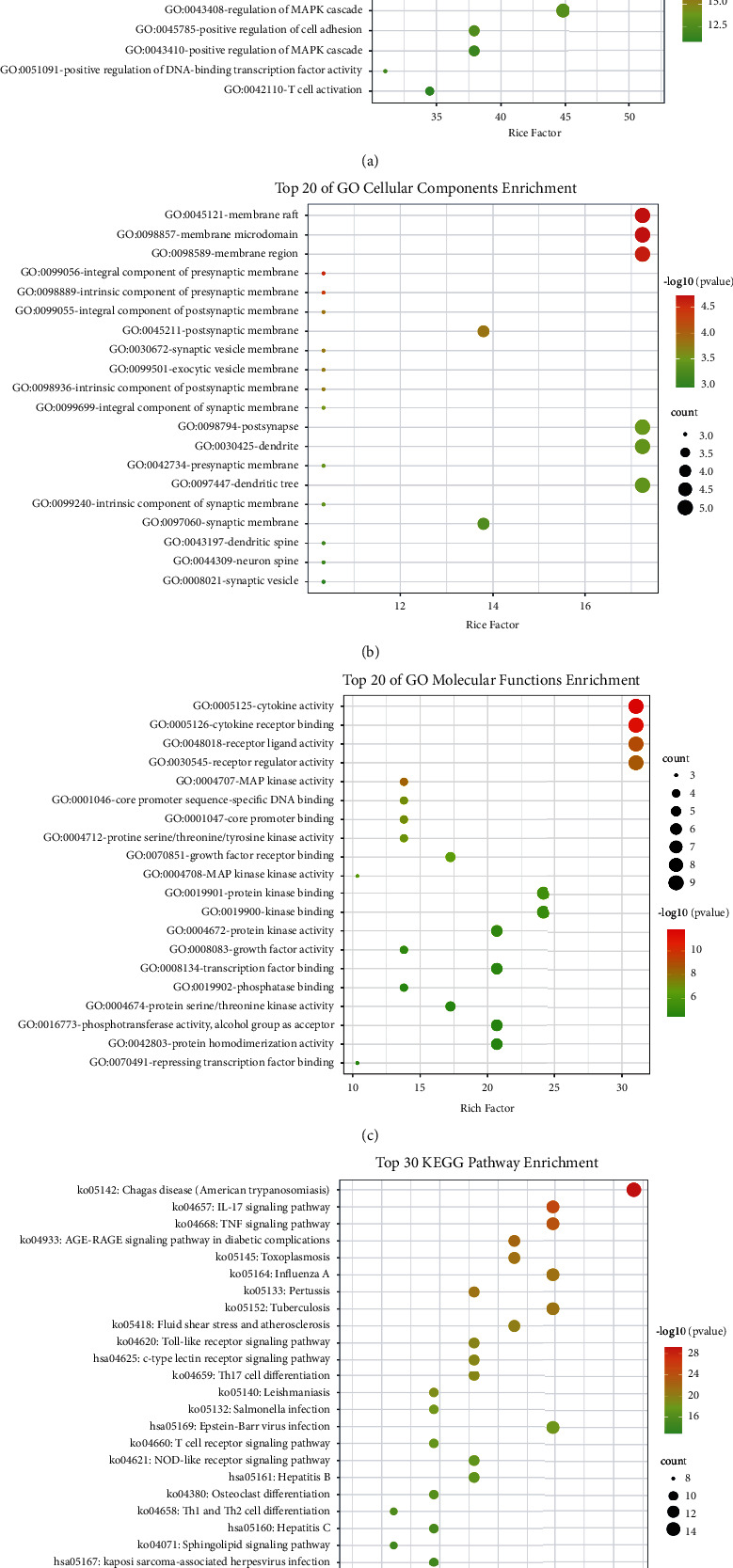
Gene ontology (GO) and Kyoto Encyclopedia of Genes and Genomes (KEGG) pathways enrichment analysis of intersecting targets of Zhisou San (ZSS) for the cough variant asthma (CVA) treatment. The top 20 terms of each part in the GO enrichment analysis are shown. GO molecular function enrichment analysis (a). GO biological process enrichment analysis (b). GO cellular components (c). KEGG pathway analysis (d). The redder the color, the more significant the genes enrichment; the sizes of the bubbles are illustrated from small to big in ascending order of the number of the intersecting targets involved in the pathways.

In order to further systematically elucidate the mechanism of ZSS in the CVA treatment, the Metascape data platform was used to analyze the signaling pathways by enriching the relevant targets. The top 20 GO items and top 30 KEGG pathways were selected based on *P* values that represent the degree of enrichment. GO analysis included three parts, namely, BP, CC, and MF. As shown in Figure 3(a), BP mainly included a response to lipopolysaccharide, response to the bacterial origin, response to biotic stimulus, and regulation of DNA-binding transcription factor activity. The top three CC were membrane raft, membrane microdomain, and membrane region (Figure 3(b)). The top three MF were cytokine activity, cytokine receptor binding, and receptor-ligand activity (Figure 3(c)). Based on the analysis of KEGG enrichment data (Figure 3(d), Table S3), the effect of ZSS was mainly correlated with inflammation and immunity such as the IL-17 signaling pathway, TNF signaling pathway, Influenza A, Pertussis, Toll-like receptor signaling pathway, and Th1 and Th2 cell differentiation.

### 3.4. Component-Target Binding Affinities Analysis

**Figure 4:**
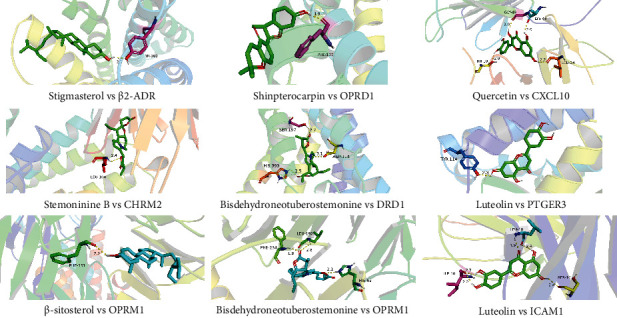
Molecular docking of active ingredients and 8 high-ranking core targets. 3D crystal structures of protein targets and the corresponding compounds ligand with the least binding energy were shown in strong affinity.

8 high-ranking core targets according to MCODE score further be predicted as the binding ability of compound to each protein using molecular docking (Table 1, Figure 4). 2 compounds showed strong affinity with *β*2-ADR, and the compound with the least binding energy was stigmasterol (-7.44 kcal/mol); 2 compounds showed strong affinity with OPRD1, and the compound with the least binding energy was shinpterocarpin (−5,78 kcal/mol); compound quercetin showed strong affinity with CXCL10 with the binding energy −6.62 kcal/mol; 11 compounds showed strong affinity with CHRM2, and the compound with the least binding energy was stemoninine B (−8.69 kcal/mol); compound bisdehydroneotuberostemonine showed strong affinity with DRD1 with the binding energy −9.38 kcal/mol; 2 compounds showed strong affinity with PTGER3, and the compound with the least binding energy was luteolin (−5.92 kcal/mol); 10 compounds showed strong affinity with OPRM1, and two compounds with the least binding energy were *β*-sitosterol (−7.35 kcal/mol) and bisdehydroneotuberostemonine (−7.35 kcal/mol); 3 compounds showed strong affinity with ICAM1, and the compound with the least binding energy was luteolin (−6.66 kcal/mol).

### 3.5. ZSS Attenuates Airway Reactivity of CVA Mice and Pathological Changes in the Lung

**Figure 5:**
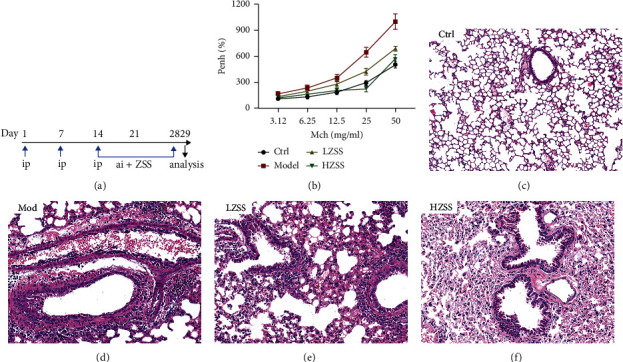
ZSS improved pulmonary function and histopathological changes. Experimental schedule (a). Airway reactivity of mice was measured by the value of calculated enhanced pause (b). Data are shown as means ± SD (*n* = 6). Representative photos of the lung from four groups (HE stained, 200× magnification, *n* = 3) (c–f). Ctrl: the control group treated with vehicle; mod: sensitization solution-induced CVA model; LZSS: low dose of ZSS treated with the model group; HZSS: high dose of ZSS treated with the model group.

To investigate the effects of ZSS on CVA in vivo, sensitization liquid was injected into the mouse tail vein and the OVA was atomized to stimulate mice for the CVA animal model shown in Figure 5(a). For airway reactivity, the airway resistance of the four groups increased with the rise of Ach concentration characterized by the value of Phen%. When treated with ZSS at low and high doses by gavage, respectively, the value of Phen% remarkably decreased compared to the saline-treated model group, and the effect was better at a high dose of ZSS (Figure 5(b)). Furthermore, H&E staining analysis of the mouse lung sections revealed that the trachea and lung tissues were intact without pathological changes in the mice from the control group (Figure 5(c)). By contrast, there were significantly pathological changes in the model group in the following manners: a large number of inflammatory cells were observed around the trachea, bronchus, and blood vessels, airway smooth muscle thickened, and goblet cell proliferation appeared (Figure 5(d)). Compared with the model group, the infiltration of inflammatory cells in lung tissue and trachea in ZSS groups were obviously reduced, and the structure of the bronchial wall was basically intact, especially in the HZSS group (Figures 5(e) and 5(f)).

### 3.6. ZSS Ameliorated Inflammatory Response in the Mouse Model of CVA

**Figure 6:**
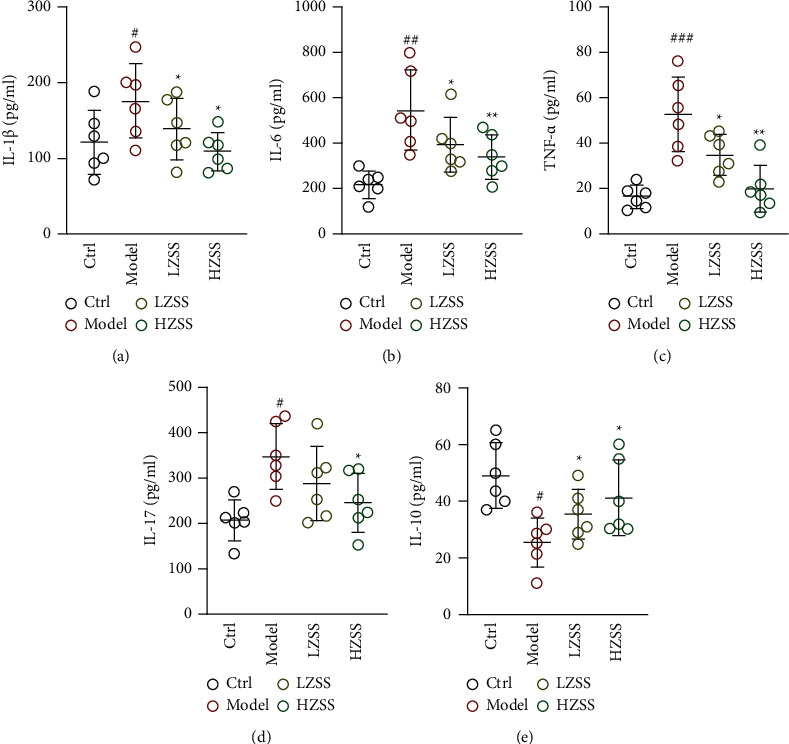
ZSS reduced inflammation in CVA mice. Levels of inflammatory cytokines in serum were evaluated by ELISA. Data are shown as means ± SD (*n* = 6). ^#^*P* < 0.05, ^##^*P* < 0.01, and ^###^*P* < 0.001 compared to the control group. ^*∗*^*P* < 0.05 and ^*∗∗*^*P* < 0.01 compared to the model group.

Cough variant asthma (CVA) is a chronic airway inflammatory disease involving multiple inflammatory mediators and inflammatory cells. To evaluate the anti-inflammatory effect of ZSS, we detected the level of cytokines IL-1*β*, IL-6, TNF-*α*, IL-17, and IL-10 in the serum of mice. As shown in Figure 6, proinflammatory factors such as IL-1*β*, IL-6, TNF-*α,* and IL-17 significantly increased in the CVA model group compared with the control group, while ZSS could effectively lower the level of these inflammatory factors (Figures 6(a)–6(d)). Conversely, the concentration of anti-inflammatory cytokine IL-10 showed significant declines in the serum of CVA mice compared to the control mice, which were ameliorated prominently in ZSS-treated CVA mice (Figure 6(e)). The level of all cytokines also well indicated that a high dose of ZSS (1.5 g/kg) might perform better than a low dose (0.75 g/kg) on CVA.

### 3.7. Effect of ZSS on the Th17/Treg Balance in the CVA Mice

**Figure 7:**
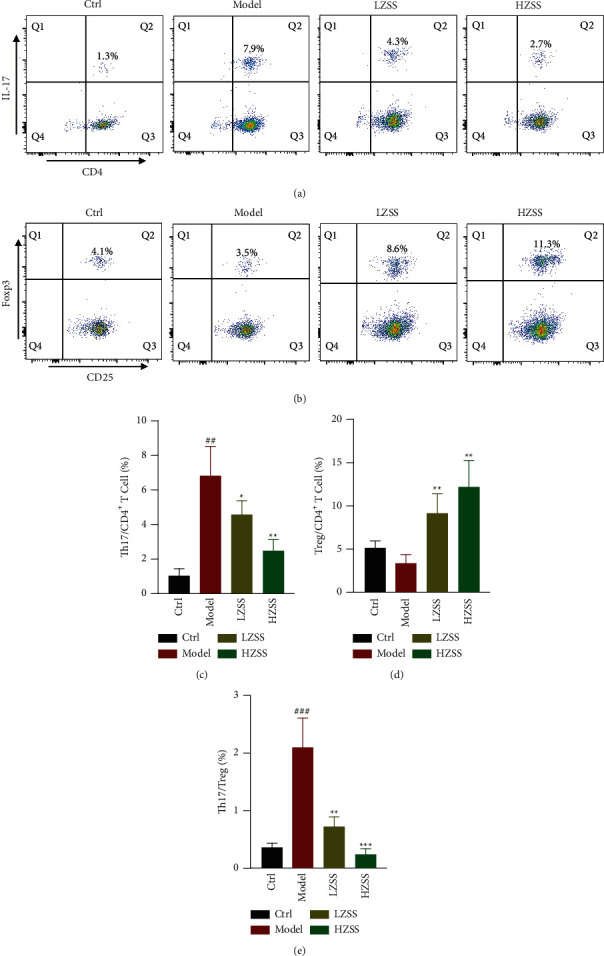
ZSS restored the balance of Th17/Treg cells in the spleen of CVA mice. Flow cytometry of Th17 in the spleens (a). Flow cytometry of Tregs in the spleens (b). Statistics of the proportion of Th17 cells (c), Treg (d), and the ratio of Th17/Treg cells (e) were analyzed. Data are shown as means ± SD (*n* = 6). ^##^*P* < 0.01 and ^###^*P* < 0.001 compared to the control group. ^*∗*^*P* < 0.05 and ^*∗∗*^*P* < 0.01 compared to the model group.

To verify the ZSS-mediated IL-17 signaling pathway and Th17 cell differentiation, we first investigated the biological effect of ZSS on the Th17/Treg balance in the mice spleen by flow cytometric analysis. The proportion of CD3^+^ CD4^+^IL-17A^+^ cells from the spleens of the CVA group was higher than that of the control group; additionally, administration of ZSS markedly reduced the percentage of Th17 cells in the spleens (Figures 7(a) and 7(c)). Treg cells exhibit immunosuppressive effects by expressing anti-inflammatory cytokines, such as IL-10. Analysis of the populations of CD4^+^CD25^+^T cells expressing Foxp3 demonstrated that the Treg cell numbers in the spleens of the ZSS-treated group were increased by 2.4- and 3.22-fold compared with those of the model group, and the Treg cell numbers in the spleens from the model group have been on a downward trajectory compared with those of the control group (Figures 7(b) and 7(d)). Moreover, we use the ratio of Th17/Treg to further illustrate ZSS-mediated Th17/Treg balance in the CVA mice. ZSS treatment obviously decreased the ratio of Th17/Treg comparable to the control mice (Figure 7(e)).

## 4. Discussion

Inhaled glucocorticoids combined with bronchodilators 2 agonists and leukotriene receptor antagonists are the mainstays of modern medical treatment for cough variant asthma (CVA). [16]. However, contraindications to treatment, side effects, or drug dependence lead to unsatisfactory results, and even though effectiveness was observed in most patients within a short time, symptoms such as cough resurfaced after quitting the medication [7]. Since cough is the only or main clinical manifestation of CVA without obvious positive lung signs, there are many clinical cases of underdiagnosis and misdiagnosis resulting in a large proportion of CVA evolving into typical asthma [2, 17]. As a result, early diagnosis and treatment may be able to prevent the disease from progressing to classic asthma.

Traditional Chinese medicine (TCM) has a long history, solid theoretical foundation, and clinical experience in the understanding of CVA, and many clinical studies have proved the definite effect on the prevention and treatment of CVA [9, 18]. According to TCM, CVA is caused by “wind evil invading lung” and “Lung Qi obstruction syndrome;” hence, the treatment should aim to expel wind to ventilate the lung as well as relieve sore throat and cough [8]. As a representative formula of TCM for treating coughs caused by wind evil invading the lung, Zhisou San (ZSS) includes ZW, BQ, and BB to relieve cough and dissolve phlegm, JG and CP to promote the lowering of Lung Qi, relieve cough, and eliminate phlegm, JJ to dispel wind and relieve symptoms, and GC to harmonize property of herbs, which has been clinically applied in the TCM treatment of CVA as various forms of single formula or combined formula [10]. Pharmacological studies have shown that ZSS has obvious effects on cough suppressing and phlegm resolving and could improve the symptoms of CVA by lowering IL-4 levels in serum and bronchoalveolar lavage fluid, increasing IFN-*γ* levels, and regulating Th1/Th2 immunological imbalance [19]. However, due to the complexity of formula components, it is still difficult to clarify the specific roles of effective substances in CVA, and some pharmacodynamic mechanism also needs to be in-depth probed. Therefore, in this study, we initially screened out major active ingredients, direct therapeutic targets, and the primary signal pathway of ZSS on CVA using the “compound-target-disease” network analysis. Furthermore, therapeutic effects were verified in vivo using an allergy-induced CVA model in pulmonary function, histopathological changes, and inflammatory response.

Combining the theoretical analysis of the compatibility of TCM prescriptions with the network pharmacology method to construct the drug-disease-target network, it was discovered that the mechanism of ZSS for the CVA treatment was not solely targeting a certain pathway or target but might work through the joint intervention of multiple targets and complex pathways, confirming the theory of the synergistic effect of herbs compatibility. Analysis results showed that 137 main chemical components of ZSS acting on 165 targets including 29 core targets played a significant role in the treatment of CVA, which were concerned with the regulation of inflammatory response and T-cell differentiation, such as IL-17 signaling pathway, TNF signaling pathway, Toll-like receptor signaling pathway, MAPK signaling pathway, T-cell receptor signaling pathway, Th17 cell differentiation, and Th1 and Th2 cell differentiation. Further analysis of the ZSS composition, we found that the targets such as *β*2-ADR, NOS2, CHRM2, DRD1, OPRM1, PTGER3, and CXCL10 in the formula were related to relaxing the broncho-gastrointestinal smooth muscle, alleviating the inflammatory response of bronchial epithelial cells and the differentiation of Th17 cells, etc., which reflected the effects of reducing phlegm and inflammation of ZW, BQ, and BB. Targets such as ICAM1, AKT1, MAPK, FOS, and BCL2 were found to serve a protective role in the immune system, demonstrating the importance of GC in enhancing immunity and harmonizing the herbs. Therefore, through the analysis of network pharmacology, the modern medical connotation of ZZS can be further explored, and the essence of the TCM alliance theory can also be further analyzed.

The multiple core targets and multiple signaling pathways predicted by the network pharmacological analysis of ZSS in the CVA treatment were essential in the pathogenesis of asthma. Asthma is a chronic inflammatory disease of the airways that involves various inflammatory cells such as T cells, eosinophils, mast cells, and neutrophils, which leads to the development of airway hyper-responsiveness [20]. Studies have shown that the pathogenesis of CVA is closely related to the imbalance in the number and function of the CD4^+^ T-cell subpopulation Th1/Th2 [21]. Among them, Th2 cells play a major role in asthma pathogenesis by secreting various cytokines such as IL-4, IL-5, IL-9, and IL-13, while IFN-*γ* secreted by Th1 inhibits Th2 function [22]. A new CD4^+^ helper T-cell subpopulation Th17 is recently identified, which produces the cytokine IL-17 that plays an important role in the development of asthma pathogenesis [23, 24]. IL-17, as an early initiator of T-cell-induced inflammatory responses, has a powerful chemotactic effect on inflammatory cells and can significantly increase inflammatory cell recruitment in the airways [25]. Activated IL-17 also activates epithelial cells, fibroblasts, and macrophages, releasing granulocyte-macrophage colony-stimulating factor (GM-CSF), TNF-*α*, PGE2, IL-1, IL-6, and IL-8, which are not only involved in airway remodeling but also promote further exacerbation of airway inflammation [23]. This suggests an important potential role of IL-17-mediated inflammation in the pathogenesis of CVA, and ZSS might be effective in treating inflammation/asthma by regulating T-cell differentiation especially IL-17 secretion after Th17 activation.

*β*2-ADR agonists are bronchial antispasmodics with wide clinical applications, which can effectively relieve the acute symptoms of asthma by selectively stimulating *β*2-ADR to relax airway smooth muscle for achieving bronchodilator effects [26]. PGE2 can directly affect the contractile and diastolic functions of airway smooth muscle cells and lead to airway remodeling in asthma by affecting the migratory growth of airway smooth muscle cells [27, 28]. In addition, DRD1 has an important role in Th17 cell differentiation and acute asthma pathogenesis [29, 30]. MAPK signaling pathway mainly regulates cell proliferation, apoptosis and differentiation, body immune and inflammatory responses, airway structural responses, and the Th1/Th2 ratio, which are involved in the formation and development of bronchial asthma [31]. MAPK signaling pathway can also be activated by DRD1 [32]. TNF-*α* is a pleiotropic proinflammatory cytokine, which promotes airway hyper-responsiveness, mucus production, various inflammatory cell activation, and phenotypes of asthma, and some studies showed that TNF-*α* as a target for asthma treatment has significant efficacy in clinical trials [33].

To further explore pharmacological effects and potential mechanisms in vivo, the mice model of CVA was established by intraperitoneal injection of ovalbumin and aluminum hydroxide as well as inhalation of ovalbumin. Sneezing, nasal rubbing, shortness of breath, abdominal muscle twitching, and other behaviors were observed in mice, which simulated well the clinical characteristics of CVA. Meanwhile, the histopathology showed that there were a large number of inflammatory cells infiltrating the trachea, bronchus, and lung tissues, and they appeared to goblet cell proliferation and smooth muscle volume increase. In addition, levels of inflammatory cytokines such as IL-1*β*, IL-6, IL-17, and TNF-*α* in the serum also increased significantly. However, ZSS could effectively relieve these changes to improve the CVA symptoms. Relevant studies have proved that the onset of CVA is not only related to the classical Th1/Th2 balance but also regulated by Th17/Treg immune balance from multiple perspectives [34, 35]. Based on the important role of inflammatory response and T-cell differentiation in the ZSS treatment of CVA, inflammatory cytokines, and Th17 cell differentiation, in particular, we furthermore found that the mechanism of ZSS in improving CVA symptoms was closely related to the regulation of Th17/Treg immune balance and related cytokines.

Studies have shown that some active ingredients of ZSS were effective in the treatment of asthma. For instance, quercetin could improve asthma response by inhibiting airway inflammation [36, 37]; kaempferol could effectively inhibit inflammation by interfering with MAPK, PI3K, and other signaling pathways to reduce the secretion of related inflammatory factors or could inhibit eosinophil infiltration of airway epithelial cells and airway inflammation by interfering with NF-*κ*B signaling [38, 39]; *β*-sitosterol might suppress asthma by inhibiting cellular response and subsequent Th2 cytokine release [40]. Our further molecular docking results revealed that stigmasterol, quercetin, stemoninine B, luteolin, and other active ingredients of ZSS had good binding with core targets related to the pathogenesis of CVA, which suggests the reliability of the ZSS previous studies from the perspective of network pharmacology, and the mechanism of multitarget and multipathway of ZSS for the treatment of CVA.

## 5. Conclusion

In summary, based on the combination of network pharmacology and experimental validation, our study proved the great clinical value of ZSS in the treatment of CVA. The findings revealed that a high dose of ZSS (1.5 g/kg/day) had critical impacts on improvements in pulmonary function, histopathology, and the inflammatory state in CVA mice, which was significantly related to regulating the Th17/Treg immune balance as the important pharmacological mechanism. However, considering the fact that ZSS contains a number of chemicals that act on multiple targets, it has certain limitations to define the active components and action targets of ZSS from the perspective of network pharmacology through the search of relevant databases, and its conclusions need to be confirmed by the clinical studies, so as to more systematically and scientifically elaborate the efficacy of ZSS in the treatment of CVA.

## Figures and Tables

**Table 1:** Eight targets with the highest score from core targets and the binding ability with active compounds of Zhisou San in treating cough variant asthma by molecular docking.

Target proteins	Symbol	Compound	Herbs	Binding energy (kcal/mol)
*β*2 Adrenergic receptor	*β*2-ADR	7-Methoxy-3-methyl-2,5-dihydroxy-9,10-dihydrophenanthrene	BB	−6.09
		Stigmasterol	JJ; BB	−7.44
Delta-type opioid receptor	OPRD1	Medicarpin	GC	−6.18
		Shinpterocarpin	GC	−5.78
C-X-C motif chemokine 10	CXCL10	Quercetin	JJ; ZW; GC	−6.62
Muscarinic acetylcholine receptor M2	CHRM2	2-Oxostenine	BB	−7.99
		Beta-sitosterol	JJ; ZW; BB; BQ	−7.68
		Bisdehydroneotuberostemonine	BB	−7.97
		Didehydrotuberostemonine	BB	−8.27
		Kaempferol	ZW; GC	−6.88
		Medicarpin	GC	−6.79
		Quercetin	JJ; ZW; GC	−5.19
		Sessilifoline B	BB	−7.15
		Stemoninine B	BB	−8.69
		Stigmasterol	JJ; BB	−7.19
		Tuberostemonine C	BB	−7.02
D1 dopamine receptor	DRD1	Bisdehydroneotuberostemonine	BB	−9.38
Prostaglandin E2 receptor EP3 subtype	PTGER3	luteolin	JJ; ZW; JG	−5.92
		Quercetin	JJ; ZW; GC	−5.12
Mu-type opioid receptor	OPRM1	7-Methoxy-2-methyl isoflavone	GC	−5.77
		7-Methoxy-3-methyl-2,5-dihydroxy-9,10-dihydrophenanthrene	BB	−6.0
		Beta-sitosterol	JJ; ZW; BB; BQ	−7.35
		Bisdehydroneotuberostemonine	BB	−7.35
		Didehydrotuberostemonine	BB	−7.52
		Inermine	GC	−6.32
		Medicarpin	GC	−5.74
		Quercetin	JJ; ZW; GC	−5.03
		Shinpterocarpin	GC	−6.97
		Tuberostemonine C	BB	−7.26
Intercellular adhesion molecule 1	ICAM1	Kaempferol	ZW; GC	−6.36
		Luteolin	JJ; ZW; JG	−6.66
		Quercetin	JJ; ZW; GC	−6.46

## Data Availability

The data used to support the findings of this study are included within the article.

## Supplementary Materials

Supplementary Materials Table S1: effective chemical components and targets of Zhisou San for the treatment of cough variant asthma Table S2: 29 core targets and associated active compounds of Zhisou San in treating cough variant asthma. Table S3: signaling pathways of the KEGG enrichment analysis in the treatment of cough variant asthma with Zhisou San.
