# Supplementary material for: Exploration in the Mechanism of Zhisou San for the Treatment of Cough Variant Asthma Based on Network Pharmacology
Source: Evid Based Complement Alternat Med. 2022 Jun 29;2022:1698571. doi: 10.1155/2022/1698571 (PMC9259218; doi:10.1155/2022/1698571)
Supplement: Supplementary Materials — Table S1: effective chemical components and targets of Zhisou San for the treatment of cough variant asthma Table S2: 29 core targets and associated active compounds of Zhisou San in treating cough variant asthma. Table S3: signaling pathways of the KEGG enrichment analysis in the treatment of cough variant asthma with Zhisou San. [file 1698571.f1.docx]

Table S1: Effective chemical components and targets of Zhisou San for the treatment of cough variant asthma

| Molecule Name | Mol ID | Drug | Target/gene |
| --- | --- | --- | --- |
| (-)-Medicocarpin | MOL004924 | gancao | PTGS2, ACHE |
| (2R)-7-hydroxy-2-(4hydroxyphenyl)chroman-4-one | MOL004941 | gancao | PTGS1, ESR1, PTGS2, ADRB2, PIK3CG, MAOB, PRKACA, CALM1 SLC6A4 |
| (2S)-2-[4-hydroxy-3-(3-methylbut-2-enyl)phenyl]-8,8-dimethyl-2,3-dihydropyrano[2,3-f]chromen-4-one | MOL004805 | gancao | CALM1，NOS2, ESR1, AR, PPARG, F10, PTGS2, ESR2, MAPK14, |
| (2S)-6-(2,4-dihydroxyphenyl)-2-(2-hydroxypropan-2-yl)-4-methoxy-2,3-dihydrofuro[3,2-g]chromen-7-one | MOL004824 | gancao | NOS2, F2, ESR1, AR, PPARG, F10, PTGS2, F7, KDR, ACHE, ESR2, DPP4, MAPK14, CDK2, CHEK1, PRSS1, CALM1 |
| (2S)-7-hydroxy-2-(4-hydroxyphenyl)-8-(3-methylbut-2-enyl)chroman-4-one | MOL004945 | gancao | NOS2, PTGS1, ESR1, SCN5A, F10, PTGS2, ADRA1B, ADRB2, ESR2, CALM1 |
| (E)-1-(2,4-dihydroxyphenyl)-3-(2,2-dimethylchromen-6-yl)prop-2-en-1-one | MOL004815 | gancao | NOS2, PTGS1, ESR1, AR, SCN5A, PPARG, F10, PTGS2, CA2, ADRA1B, ESR2, MAPK14, CDK2, CHEK1, CALM1 |
| (E)-3-[3,4-dihydroxy-5-(3-methylbut-2-enyl)phenyl]-1-(2,4-dihydroxyphenyl)prop-2-en-1-one | MOL004898 | gancao | ESR1, AR, PPARG, PTGS2, MAPK14, CDK2, CALM1 |
| 1,3-dihydroxy-8,9-dimethoxy-6-benzofurano[3,2-c]chromenone | MOL004914 | gancao | ESR1, AR, PPARG, MAPK14, CDK2, CHEK1, PRKACA |
| 1,3-dihydroxy-9-methoxy-6-benzofurano[3,2-c]chromenone | MOL004913 | gancao | ESR1, PPARG, ESR2, MAPK14, CDK2, CHEK1, PRKACA |
| 16beta,17-dihydroxy-(-)-kauran-19-ate-beta-D-glucose ester_qt | MOL010428 | ziwan | NR3C2 |
| 1-Methoxyphaseollidin | MOL004959 | gancao | NOS2, PTGS1, F2, ESR1, AR, SCN5A, PPARG, F10, PTGS2, NOS3, KDR, ADRA1B, ADRB2, ESR2, MAPK14, CDK2, PIK3CG, PRSS1, CALM1 |
| 2-(3,4-dihydroxyphenyl)-5,7-dihydroxy-6-(3-methylbut-2-enyl)chromone | MOL004866 | gancao | F2, AR, SCN5A, PPARG, F10, PTGS2, F7, ADRB2, DPP4, CDK2, CHEK1, PRSS1, CALM1 |
| 2-[(3R)-8,8-dimethyl-3,4-dihydro-2H-pyrano[6,5-f]chromen-3-yl]-5-methoxyphenol | MOL004978 | gancao | NOS2, PTGS1, CHRM3, CHRM1, ESR1, AR, SCN5A, PPARG, F10, PTGS2, NOS3, ACHE, ADRA1B, SLC6A3, ADRB2, ESR2, MAPK14, CDK2, CHEK1, PRKACA, PRSS1, KCNMA1, CALM1 |
| 2-oxostenine | MOL009379 | baibu | AR, PTGS2, CHRM2 |
| 3-(2,4-dihydroxyphenyl)-8-(1,1-dimethylprop-2-enyl)-7-hydroxy-5-methoxy-coumarin | MOL004849 | gancao | NOS2, F2, ESR1, AR, PPARG, F10, PTGS2, F7, KDR, ESR2, DPP4, MAPK14, CDK2, CHEK1, PRSS1, CALM1 |
| 3-(3,4-dihydroxyphenyl)-5,7-dihydroxy-8-(3-methylbut-2-enyl)chromone | MOL004863 | gancao | NOS2, F2, ESR1, AR, PPARG, F10, PTGS2, MAPK14, CDK2, CHEK1, PRSS1, CALM1 |
| 3,3'-bis-(3,4-dihydro-4-hydroxy-6-methoxy)-2H-1-benzopyran | MOL009386 | baibu | ESR1, PTGS2, ADRB2, CALM1 |
| 3'-Hydroxy-4'-O-Methylglabridin | MOL004966 | gancao | NOS2, PTGS1, ESR1, AR, SCN5A, PPARG, F10, PTGS2, F7, KDR, ADRA1B, ADRB2, ESR2, MAPK14, CDK2, CHEK1, PRKACA, PRSS1, KCNMA1, CALM1 |
| 3'-Methoxyglabridin | MOL004974 | gancao | NOS2, PTGS1, ESR1, AR, SCN5A, PPARG, F10, PTGS2, F7, ACHE, ADRA1B, ADRB2, ESR2, MAPK14, CDK2, CHEK1, PRSS1, KCNMA1, CALM1 |
| 5,7-dihydroxy-2-(3-hydroxy-4-methoxyphenyl)chroman-10-one | MOL000359 | jingjie, ziwan, baibu | F2, PIK3CG |
| 5,7-dihydroxy-2-(3-hydroxy-4-methoxyphenyl)chroman-11-one | MOL000359 | jingjie | SOD1 |
| 5,7-dihydroxy-2-(3-hydroxy-4-methoxyphenyl)chroman-12-one | MOL000359 | jingjie | MMP3 |
| 5,7-dihydroxy-2-(3-hydroxy-4-methoxyphenyl)chroman-4-one | MOL000359 | ziwan, baibu, chenpi, jingjie, baibu | JUN, PTGS1, PTGS2, PRKACA, CALM1, SCN5A, PIK3CG |
| 5,7-dihydroxy-2-(3-hydroxy-4-methoxyphenyl)chroman-6-one | MOL000359 | Jingjie | JUN |
| 5,7-dihydroxy-2-(3-hydroxy-4-methoxyphenyl)chroman-7-one | MOL000359 | jingjie, ziwan, baibu | PLAT, PRKACA |
| 5,7-dihydroxy-2-(3-hydroxy-4-methoxyphenyl)chroman-8-one | MOL000359 | jingjie, ziwan, baibu | F3, CALM1 |
| 5,7-dihydroxy-2-(3-hydroxy-4-methoxyphenyl)chroman-9-one | MOL000359 | jingjie, ziwan, baibu | THBD, SCN5A |
| 5,7-dihydroxy-3-(4-methoxyphenyl)-8-(3-methylbut-2-enyl)chromone | MOL004864 | gancao | NOS2, ESR1, AR, PPARG, F10, PTGS2, ESR2, DPP4, MAPK14, CDK2, CHEK1, PRSS1, CALM1 |
| 6-prenylated eriodictyol | MOL004989 | gancao | NOS2, ESR1, SCN5A, F10, PTGS2, F7, CALM1 |
| 7,2',4'-trihydroxy－5-methoxy-3－arylcoumarin | MOL004990 | gancao | NOS2, PTGS1, ESR1, AR, PPARG, PTGS2, ESR2, DPP4, MAPK14, CDK2, CHEK1, PRKACA |
| 7-Acetoxy-2-methylisoflavone | MOL004991 | gancao | NOS2, PTGS1, F2, ESR1, AR, SCN5A, PPARG, PTGS2, NOS3, ACHE, ADRA1B, ADRB2, DPP4, MAPK14, CDK2, CHEK1, PRSS1, CALM1 |
| 7-Methoxy-2-methyl isoflavone | MOL003896 | gancao | NOS2, PTGS1, DRD1, CHRM3, F2, CHRM1, ESR1, AR, ADRB1, SCN5A, PPARG, PTGS2, ACHE, ADRA1B, SLC6A3, ADRB2, SLC6A4, ESR2, DPP4, MAPK14, CDK2, LTA4H, MAOB, CHRNA7, CHEK1, PRKACA, PRSS1, CALM1, NOS3, OPRM1 |
| 7-methoxy-3-methyl-2,5-dihydroxy-9,10-dihydrophenanthrene | MOL009374 | baibu | NOS2, PTGS1, DRD1, CHRM3, F2, CHRM1, ESR1, AR, SCN5A, PTGS2, β2-ADR, ACHE, HTR2A, ADRA1B, SLC6A3, ADRB2, SLC6A4, OPRM1, ESR2, DPP4, MAPK14, CDK2, LTA4H, PRKACA |
| 8-(6-hydroxy-2-benzofuranyl)-2,2-dimethyl-5-chromenol | MOL004838 | gancao | NOS2, ESR1, PTGS2, PIK3CG |
| 8-prenylated eriodictyol | MOL004993 | gancao | ESR1, SCN5A, F10, PTGS2, F7, CALM1 |
| acacetin | MOL001689 | jiegeng | NOS2, PTGS1, AR, PTGS2, DPP4, CDK2, PRKACA, PRSS1, CALM1, PIK3CG, CHEK1, ADRB2, RELA, BCL2, CDKN1A, BAX, CASP3, TP53, CASP8, FASLG |
| astin C | MOL010455 | ziwan | F10 |
| beta-sitosterol | MOL000358 | jingjie | COL3A1, COL1A1, F10, F7, TP53, CDK4, CD40LG, CCL2, CAV1, CASP9, CASP8, CASP3, CA2, CALM1, ADRB2, ADRB1, BCL2L1, BIRC5, PGR, PTGS1, PTGS2, PIK3CG, PRKACA, DRD1, CHRM3, CHRM1, SCN5A, GABRA2, HTR2A, CHRM2, ADRA1B, SLC6A4, OPRM1, CHRNA7, BCL2, BAX, JUN, PRKCA, TGFB1, PON1 |
| bisdehydroneotuberostemonine | MOL009377 | baibu | NOS2, DRD1, CHRM3, F2, CHRM1, AR, PTGS2, CHRM2, ADRA1B, ADRB2, DRD2, OPRM1, CHRNA7 |
| bisdehydrostemoninine | MOL009380 | baibu | AR, PTGS2 |
| bisdehydrostemoninine A | MOL009381 | baibu | AR, PTGS2 |
| bisdehydrostemoninine B | MOL009382 | baibu | NOS2, AR, PTGS2 |
| Calycosin | MOL000417 | gancao | NOS2, PTGS1, ESR1, AR, PPARG, PTGS2, ESR2, DPP4, MAPK14, CDK2, CHEK1, PRKACA, PRSS1, CALM1, ADRB2 |
| cis-Dihydroquercetin | MOL004580 | jiegeng | PTGS1, PTGS2, PIK3CG, AKR1B1 |
| Citromitin | MOL005815 | chenpi | SCN5A, F10, PTGS2, F7, KCNMA1, CALM1 |
| dehydroglyasperins C | MOL005020 | gancao | NOS2, ESR1, AR, SCN5A, PPARG, F10, PTGS2, ADRB2, ESR2, MAPK14, CDK2, CHEK1, PRSS1, CALM1 |
| DFV | MOL001792 | gancao | PTGS1, ESR1, PTGS2, ADRB2, PIK3CG, PRKACA, MAOB, SLC6A4 |
| Didehydrotuberostemonine | MOL009387 | baibu | CHRM3, F2, CHRM1, AR, SCN5A, PTGS2, CHRM2, ADRA1B, OPRM1, DPP4 |
| Dihydrostemoninine | MOL009388 | baibu | AR |
| Diosmetin | MOL002881 | jingjie | TNF, PRSS1, TGFB1, RELA |
| euchrenone | MOL004806 | gancao | NOS2, ESR1, SCN5A, F10, PTGS2, ESR2, CALM1 |
| Eurycarpin A | MOL004915 | gancao | NOS2, F2, ESR1, AR, SCN5A, PPARG, F10, PTGS2, ESR2, DPP4, MAPK14, CDK2, CHEK1, PRSS1, CALM1 |
| formononetin | MOL000392 | baibu, gancao | NOS2, PTGS1, CHRM1, ESR1, AR, PPARG, PTGS2, SLC6A3, ADRB2, SLC6A4, ESR2, DPP4, MAPK14, CDK2, MAOB, CHEK1, PRKACA, PRSS1, CALM1, F2, NOS3, ACHE, JUN, IL4 |
| galangin | MOL002563 | ziwan | NOS2, PTGS1, AR, PPARG, PTGS2, DPP4, PIK3CG, CHEK1, PRKACA, BCL2, CDK4, CYP1A1, GSTP1, AHR |
| Gancaonin A | MOL004856 | gancao | NOS2, F2, ESR1, AR, SCN5A, PPARG, F10, PTGS2, ACHE, ESR2, DPP4, CHEK1, PRSS1, CALM1 |
| Gancaonin B | MOL004857 | gancao | NOS2, F2, ESR1, AR, PPARG, F10, PTGS2, F7, KDR, ADRA1B, ADRB2, ESR2, DPP4, CHEK1, PRSS1, CALM1 |
| Gancaonin G | MOL005000 | gancao | NOS2, F2, ESR1, AR, PPARG, F10, PTGS2, NOS3, ESR2, DPP4, MAPK14, CHEK1, PRSS1, CALM1 |
| Gancaonin H | MOL005001 | gancao | ESR1, AR, F10, PTGS2, KDR, PRSS1, CALM1 |
| Glabranin | MOL004910 | gancao | NOS2, PTGS1, ESR1, SCN5A, F10, PTGS2, NOS3, PRKACA, CALM1 |
| Glabrene | MOL004911 | gancao | NOS2, PTGS1, ESR1, AR, SCN5A, PPARG, F10, PTGS2, ADRB2, ESR2, MAPK14, CDK2, PRSS1, CALM1 |
| Glabridin | MOL004908 | gancao | NOS2, CHRM1, ESR1, AR, SCN5A, PPARG, PTGS2, ACHE, ADRA1B, ADRB2, ESR2, MAPK14, CDK2, CHEK1, PRKACA, PRSS1, CALM1 |
| Glabrone | MOL004912 | gancao | NOS2, PTGS1, F2, ESR1, AR, SCN5A, PPARG, F10, PTGS2, ACHE, ESR2, DPP4, MAPK14, CDK2, CHEK1, PRSS1, CALM1 |
| glaucogenin,a | MOL006919 | baiqian | NR3C2, NR3C1, PTGS2 |
| Glepidotin A | MOL004828 | gancao | NOS2, PTGS1, F2, ESR1, AR, SCN5A, PPARG, F10, PTGS2, NOS3, F7, KDR, DPP4, MAPK14, CDK2, CHEK1, PRSS1, CALM1 |
| Glepidotin B | MOL004829 | gancao | PTGS1, ESR1, SCN5A, F10, PTGS2, NOS3, F7, ADRA1B, CALM1, |
| Glyasperin C | MOL004811 | gancao | NOS2, F2, ESR1, AR, SCN5A, PPARG, F10, PTGS2, ACHE, ESR2, DPP4, MAPK14, CDK2, CHEK1, PRSS1, CALM1 |
| glyasperin F | MOL004810 | gancao | NOS2, PTGS1, ESR1, AR, SCN5A, PPARG, F10, PTGS2, ESR2, MAPK14, CDK2, PRSS1, CALM1 |
| Glyasperins M | MOL005007 | gancao | NOS2, PTGS1, ESR1, AR, SCN5A, PPARG, F10, PTGS2, F7, KDR, ACHE, ESR2, CDK2, PRKACA, PRSS1, KCNMA1, CALM1 |
| Glycyrin | MOL004879 | gancao | NOS2, F2, ESR1, AR, PPARG, F10, PTGS2, KDR, ESR2, DPP4, CHEK1, PRSS1, CALM1 |
| Glycyrrhiza flavonol A | MOL005008 | gancao | NOS2, ESR1, AR, F10, PTGS2, F7, ACHE, ESR2, DPP4, CDK2, PRSS1, CALM1 |
| Glypallichalcone | MOL004835 | gancao | NOS2, PTGS1, CHRM1, ESR1, AR, SCN5A, PPARG, PTGS2, CA2, ADRA1B, SLC6A3, ADRB2, SLC6A4, ESR2, MAPK14, CDK2, LTA4H, MAOB, CHEK1, PRKACA, CALM1 |
| Glyzaglabrin | MOL004907 | gancao | NOS2, PTGS1, ESR1, AR, PPARG, PTGS2, ESR2, DPP4, MAPK14, CDK2, PIK3CG, CHEK1, PRKACA, PRSS1 |
| HMO | MOL004957 | gancao | NOS2, PTGS1, CHRM1, ESR1, AR, SCN5A, PPARG, PTGS2, SLC6A3, ADRB2, SLC6A4, ESR2, DPP4, MAPK14, CDK2, MAOB, CHEK1, PRKACA, PRSS1, CALM1 |
| Inermine | MOL001484 | gancao | PTGS1, CHRM3, SCN5A, PTGS2, HTR3A, ADRA1B, PIK3CG, PRKACA, PRSS1, CALM1, CHRM1, ADRB2, OPRM1 |
| Inflacoumarin A | MOL004980 | gancao | F2, ESR1, AR, PPARG, F10, PTGS2, ADRB2, DPP4, PRSS1, CALM1, PTGS1, SCN5A |
| Isoglycyrol | MOL004948 | gancao | NOS2, ESR1, AR, PTGS2, DPP4 |
| Isolicoflavonol | MOL004949 | gancao | NOS2, F2, ESR1, AR, PPARG, F10, PTGS2, CDK2, PRSS1, CALM1 |
| isorhamnetin | MOL000354 | ziwan, gancao | NOS2, PTGS1, ESR1, AR, PPARG, PTGS2, ESR2, DPP4, MAPK14, CDK2, PIK3CG, PRKACA, PRSS1, CALM1, CHEK1, AKR1B1, F7, F2, NOS3, ACHE, MAOB, RELA |
| Isotrifoliol | MOL004814 | gancao | NOS2, ESR1, AR, PTGS2, ESR2, MAPK14, CDK2, PIK3CG, CHEK1, PRKACA |
| Jaranol | MOL000239 | gancao | NOS2, PTGS1, AR, SCN5A, PTGS2, ESR2, DPP4, CDK2, CHEK1, PRSS1, CALM1 |
| kaempferol | MOL000422 | ziwan, gancao | NOS2, PTGS1, AR, PPARG, PTGS2, PIK3CG, PRKACA, DPP4, PRSS1, PGR, F2, CHRM1, NOS3, GABRA2, ACHE, SLC6A2, CHRM2, ADRA1B, F7, CALM1, RELA, IKBKB, AKT1, BCL2, BAX, TNF, JUN, CASP3, MAPK8, MMP1, STAT1, HMOX1, CYP3A4, CYP1A2, CYP1A1, ICAM1, SELE, VCAM1, NR1I2, CYP1B1, ALOX5, GSTP1, AHR, INSR, GSTM1, SLPI |
| Kanzonol F | MOL004988 | gancao | ESR1, AR, F10, PTGS2, ESR2, CALM1 |
| kanzonols W | MOL004820 | gancao | NOS2, PTGS1, ESR1, AR, SCN5A, PPARG, F10, PTGS2, ESR2, MAPK14, CDK2, CHEK1, PRSS1, CALM1 |
| Licoagrocarpin | MOL005003 | gancao | NOS2, PTGS1, CHRM3, F2, CHRM1, ESR1, AR, SCN5A, PPARG, F10, PTGS2, NOS3, ACHE, ADRA1B, ADRB2, ESR2, MAPK14, CDK2, PRSS1, CALM1 |
| Licoagroisoflavone | MOL005012 | gancao | NOS2, F2, ESR1, AR, SCN5A, PPARG, F10, PTGS2, ESR2, DPP4, MAPK14, CDK2, CHEK1, PRSS1, CALM1 |
| licochalcone A | MOL000497 | gancao | NOS2, PTGS1, CHRM1, ESR1, AR, SCN5A, PPARG, F10, PTGS2, CA2, ADRA1B, SLC6A3, ESR2, MAPK14, CDK2, CHEK1, CALM1, ADRB2, RELA, STAT3, CCND1, BCL2, MAPK1, RB1, CDK4 |
| Licochalcone B | MOL004841 | gancao | NOS2, PTGS1, ESR1, AR, PPARG, PTGS2, CA2, ADRB2, ESR2, MAPK14, CDK2, CHEK1, PRKACA, CALM1 |
| licochalcone G | MOL004848 | gancao | NOS2, ESR1, AR, PPARG, F10, PTGS2, KDR, ESR2, MAPK14, CDK2, CALM1 |
| Licocoumarone | MOL004882 | gancao | ESR1, AR, ESR2, CDK2 |
| Licoisoflavone | MOL004883 | gancao | NOS2, F2, ESR1, AR, PPARG, F10, PTGS2, KDR, DPP4, MAPK14, CDK2, CHEK1, PRSS1, CALM1 |
| Licoisoflavone B | MOL004884 | gancao | NOS2, NOS2, F2, ESR1, AR, PPARG, F10, PTGS2, ACHE, ESR2, CDK2, CHEK1, PRSS1, CALM1 |
| licopyranocoumarin | MOL004904 | gancao | NOS2, F2, ESR1, AR, PPARG, F10, PTGS2, F7, KDR, ACHE, CDK2, PRSS1, CALM1 |
| Licoricone | MOL004855 | gancao | NOS2, F2, ESR1, AR, PPARG, F10, PTGS2, KDR, CHEK1, PRSS1, CALM1 |
| liquiritin | MOL004903 | gancao | F10, F7, CALM1, PTGS2, KDR, SOD1 |
| Lupiwighteone | MOL003656 | gancao | NOS2, F2, ESR1, AR, SCN5A, PPARG, F10, PTGS2, ESR2, DPP4, MAPK14, CDK2, CHEK1, PRSS1, CALM1 |
| luteolin | MOL000006 | jiegeng, jingjie, ziwan | PTGS1, AR, PTGS2, PRSS1, PRKACA, PIK3CG, RELA, EGFR, AKT1, VEGFA, CCND1, BCL2L1, CDKN1A, CASP9, MMP2, MMP9, MAPK1, IL10, RB1, CDK4, TNF, JUN, IL6, CASP3, TP53, NFKBIA, MDM2, MMP1, PCNA, ERBB2, PPARG, HMOX1, ICAM1, BIRC5, IL2, TYR, IFNG, IL4, GSTP1, INSR, CD40LG, PTGES, MET, SLC6A4, SLC6A2, SLC6A3, SCN5A, STAT1, PON1, CHEK2, ERBB3, RAF1, FOS, PRKCA, PTGER3, PGR, EGF, DPP4 |
| Mairin | MOL000211 | gancao | PGR |
| Medicarpin | MOL002565 | gancao | NOS2, PTGS1, DRD1, CHRM3, CHRM1, ESR1, SCN5A, PTGS2, HTR2A, CHRM2, ADRA1B, SLC6A3, ADRB2, SLC6A4, OPRM1, ESR2, DPP4, MAPK10, CDK2, PIK3CG, CHRNA7, PRKACA, PRSS1, CALM1, OPRD1 |
| naringenin | MOL004328 | chenpi, gancao | PTGS1, ESR1, PTGS2, PRKACA, PIK3CG, RELA, AKT1, BCL2, MAPK3, MAPK1, CASP3, LDLR, SOD1, CAT, PPARG, APOB, HMGCR, GSTP1, UGT1A1, PPARA, GSR, ABCC1, ADIPOQ, CES1 |
| nobiletin | MOL005828 | chenpi | CALM1, SCN5A, BCL2, BAX, CASP9, MMP9, JUN, TP53, MAPK8, TIMP1, PPARG, CREB1, PLA2G4A |
| Odoratin | MOL005016 | gancao | NOS2, PTGS1, ESR1, AR, SCN5A, PPARG, PTGS2, ESR2, DPP4, MAPK14, CDK2, CHEK1, PRSS1, CALM1 |
| oxystemoninine | MOL009409 | baibu | AR |
| Phaseol | MOL005017 | gancao | F2, ESR1, AR, PPARG, PTGS2, KDR, MAPK14, CDK2, CHEK1, PRKACA |
| Phaseolinisoflavan | MOL004833 | gancao | NOS2, CHRM1, ESR1, AR, SCN5A, PPARG, F10, PTGS2, ACHE, ADRA1B, ADRB2, ESR2, MAPK14, CDK2, CHEK1, PRSS1, CALM1 |
| protostemotinine | MOL009411 | baibu | NR3C2, NR3C1 |
| quercetin | MOL000098 | jingjie, ziwan, gancao | PARP1, SERPINE1, PIK3CG, PPARG, PPARA, SPP1, ODC1, NR1I2, NFE2L2, NOS3, NOS2, NFKBIA, CHRNA7, POR, MPO, MYC, OPRM1, CHRM3, CHRM2, CHRM1, PRKACA, MAPK1, NR3C2, MMP9, LTA4H, MMP1, CXCL8, IL6, IL4, IL2, IL10, IL1B, IL1A, IRF1, IFNG, ICAM1, IGFBP3, IGF2, INSR, CHUK, HIF1A, MET, HMOX1, HSPB1, GSTP1, GSTM1, GJA1, GABRA2, CCND1, SELE, EGFR, MDM2, DPP4, CYP3A4, CYP1B1, CYP1A2, CYP1A1, CDKN2A, CDKN1A, CXCL10, PTGS1, AR, PTGS2, AKR1B1, PRSS1, F2, SCN5A, F10, ADRB2, MMP3, F7, ACHE, MAOB, RELA, AKT1, VEGFA, BCL2, BCL2L1, FOS, BAX, CASP9, PLAU, MMP2, EGF, RB1, TNF, JUN, CASP3, TP53, CASP8, RAF1, SOD1, PRKCA, STAT1, ERBB2, CAV1, F3, CCL2, VCAM1, PTGER3, BIRC5, TGFB1, PLAT, THBD, COL1A1, ALOX5, ABCA2, AHR, COL3A1, CHEK2, CD40LG, ERBB3, PON1 |
| Quercetin der. | MOL004961 | gancao | NOS2, PTGS1, ESR1, AR, SCN5A, PPARG, PTGS2, ESR2, DPP4, MAPK14, CDK2, PRSS1, CALM1 |
| rabdosinatol | MOL010470 | ziwan | NR3C2 |
| Schkuhrin I | MOL011849 | jingjie | VEGFA, VCAM1, PLAU, TYR |
| Semilicoisoflavone B | MOL004827 | gancao | NOS2, F2, ESR1, AR, SCN5A, PPARG, F10, PTGS2, F7, ACHE, CDK2, CHEK1, PRSS1, CALM1 |
| sesamin | MOL001558 | baibu | F10, PTGS2, SCN5A, CCND1, IL10, G6PD, NOS3, ECE1, CYP2B6, UGT1A1, NOX1 |
| sessilifoliamide C | MOL009414 | baibu | PTGS2 |
| sessilifoliamide H | MOL009419 | baibu | AR |
| sessilifoline B | MOL009422 | baibu | CHRM2, CHRNA7 |
| sessilistemonamine A | MOL009423 | baibu | AR, NR3C1 |
| sessilistemonamine B | MOL009424 | baibu | AR, NR3C1 |
| shinpterocarpin | MOL004891 | gancao | NOS2, PTGS1, CHRM3, CHRM1, ESR1, AR, SCN5A, PPARG, PTGS2, OPRD1, ADRA1B, ADRB2, OPRM1, ESR2, MAPK14, CDK2, PIK3CG, CHRNA7, PRKACA, PRSS1, CALM1 |
| shionone | MOL010473 | ziwan | PGR |
| Sigmoidin-B | MOL004935 | gancao | ESR1, F10, PTGS2, KDR, CALM1 |
| sitosterol | MOL000359 | jingjie, baibu, baiqian,chenpi, gancao, jiegeng, ziwan | RELA, PGR, NR3C2 |
| stemonamide | MOL009430 | baibu | AR, PTGS2, NR3C2, NR3C1 |
| stemonine | MOL009431 | baibu | AR |
| stemoninine B | MOL009433 | baibu | NOS2, CHRM3, CHRM1, AR, PTGS2, CHRM2, ADRA1B |
| stemoninoamide | MOL009434 | baibu | AR, PTGS2 |
| Stigmasterol | MOL000449 | jingjie, baibu | ABCA2, AHR, ALOX5, BCL2, BAX, AR, MAOB, MAOA, β2-ADR, ADRA1B, AKR1B1, ADH1C, ACHE, MMP2, HTR2A, PGR, NR3C2, PTGS1, PTGS2, SLC6A2, SLC6A3, ADRB2, PLAU, LTA4H, PRKACA, CHRM3, CHRM1, ADRB1, SCN5A, CHRM2, CHRNA7 |
| suchilactone | MOL005384 | baibu | SCN5A, F10, PTGS2, F7, ADRB2, KCNMA1, CALM1, PTGS1, PRKACA |
| tuberostemonine C | MOL009363 | baibu | AR, CHRM2, NR3C2, OPRM1 |
| Tylophorinidine | MOL012933 | baiqian | PTGS2 |
| Vestitol | MOL000500 | gancao | NOS2, PTGS1, CHRM1, ESR1, AR, SCN5A, PPARG, PTGS2, HTR2A, ADRA1B, SLC6A3, ADRB2, SLC6A4, ESR2, DPP4, MAPK14, CDK2, CHEK1, PRKACA, PRSS1, CALM1 |
| Xambioona | MOL005018 | gancao | NOS2, ESR1, F10, PTGS2, ESR2, CALM1 |
| ZINC03978781 | MOL003036 | ziwan | PGR |
| Spinasterol | MOL004355 | jiegeng, ziwan | PGR, NR3C2 |
| 5,7-dihydroxy-2-(3-hydroxy-4-methoxyphenyl)chroman-5-one | MOL000359 | jingjie, ziwan, baibu | JUN, PTGS2 |
| ZINC03978783 | MOL003036 | ziwan | NR3C2 |
| Glycyrol | MOL004948 | gancao | NOS2, ESR1, PPARG, PTGS2, KDR, MAPK14, CHEK1, F2 |
| glyasperin B | MOL004808 | gancao | NOS2, F2, ESR1, AR, PPARG, F10, PTGS2, F7, FLT1, ACHE, ESR2, DPP4, CDK2, PRSS1, CALM1 |
| licoisoflavanone | MOL004885 | gancao | NOS2, PTGS1, ESR1, AR, SCN5A, PPARG, F10, PTGS2, F7, ACHE, ESR2, CDK2, PRSS1, CALM1 |

Table S2: 29 core targets and associated active compounds of Zhisou San in treating cough variant asthma.

| Molecule Name | MOL ID | Drug | Target/gene |
| --- | --- | --- | --- |
| acacetin | MOL001689 | jiegeng | NOS2, RELA, BCL2 |
| luteolin | MOL000006 | Jiegeng, jingjie, ziwan | RELA, AKT1, BCL2L1, IL10, TNF, IL6, ICAM1, IL2, IFNG, IL4, FOS, PTGER3 |
| Diosmetin | MOL002881 | jingjie | TNF, RELA |
| sitosterol | MOL000358 | jingjie, ziwan, baibu, baiqian | RELA, CCL2, BCL2L1, CHRM2, OPRM1, BCL2 |
| quercetin | MOL000098 | jingjie,ziwan, gancao | NOS2, MYC, OPRM1, CHRM2, IL6, IL4, IL2, IL10, IL1B, IFNG, ICAM1, CXCL10, RELA, AKT1, BCL2, BCL2L1, FOS, TNF, CCL2, PTGER3 |
| beta-sitosterol | MOL000358 | jingjie, ziwan, baibu, baiqian | CCL2, BCL2L1, CHRM2, OPRM1, BCL2 |
| Stigmasterol | MOL000449 | jingjie, baibu | BCL2, β2-ADR, CHRM2 |
| galangin | MOL002563 | ziwan | NOS2, BCL2 |
| isorhamnetin | MOL000354 | ziwan, gancao | NOS2, ESR1, MAPK14, RELA |
| kaempferol | MOL000422 | ziwan, gancao | NOS2, CHRM2, RELA, AKT1, BCL2, TNF, MAPK8, ICAM1 |
| sesamin | MOL001558 | baibu | IL10 |
| formononetin | MOL000392 | baibu, gancao | NOS2, ESR1, MAPK14, IL4 |
| tuberostemonine C | MOL009363 | baibu | CHRM2, OPRM1 |
| 7-methoxy-3-methyl-2,5-dihydroxy-9,10-dihydrophenanthrene | MOL009374 | baibu | NOS2, ESR1, β2-ADR, OPRM1, MAPK14 |
| bisdehydroneotuberostemonine | MOL009377 | baibu | NOS2, CHRM2, DRD1, OPRM1 |
| 2-oxostenine | MOL009379 | baibu | CHRM2 |
| bisdehydrostemoninine B | MOL009382 | baibu | NOS2 |
| 3,3'-bis-(3,4-dihydro-4-hydroxy-6-methoxy)-2H-1-benzopyran | MOL009386 | baibu | ESR1 |
| didehydrotuberostemonine | MOL009387 | baibu | CHRM2, OPRM1 |
| protostemotinine | MOL009411 | baibu | NR3C1 |
| sessilifoline B | MOL009422 | baibu | CHRM2 |
| sessilistemonamine A | MOL009423 | baibu | NR3C1 |
| sessilistemonamine B | MOL009424 | baibu | NR3C1 |
| stemonamide | MOL009430 | baibu | NR3C1 |
| stemoninine B | MOL009433 | baibu | NOS2, CHRM2 |
| glaucogenin,a | MOL006919 | baiqian | NR3C1 |
| naringenin | MOL004328 | chenpi, gancao | ESR1, RELA, AKT1, BCL2, MAPK3 |
| nobiletin | MOL005828 | chenpi | BCL2, MAPK8 |
| Inermine | MOL001484 | gancao | OPRM1 |
| DFV | MOL001792 | gancao | ESR1 |
| Glycyrol | MOL002311 | gancao | NOS2, ESR1, MAPK14 |
| Jaranol | MOL000239 | gancao | NOS2 |
| Medicarpin | MOL002565 | gancao | NOS2, ESR1, CHRM2, OPRM1, MAPK10, OPRD1 |
| Lupiwighteone | MOL003656 | gancao | NOS2, ESR1, MAPK14 |
| 7-Methoxy-2-methyl isoflavone | MOL003896 | gancao | NOS2, ESR1, MAPK14, OPRM1 |
| Calycosin | MOL000417 | gancao | NOS2, ESR1, MAPK14 |
| (2S)-2-[4-hydroxy-3-(3-methylbut-2-enyl)phenyl]-8,8-dimethyl-2,3-dihydropyrano[2,3-f]chromen-4-one | MOL004805 | gancao | NOS2, ESR1, MAPK14 |
| euchrenone | MOL004806 | gancao | NOS2, ESR1 |
| glyasperin B | MOL004808 | gancao | NOS2, ESR1 |
| glyasperin F | MOL004810 | gancao | NOS2, ESR1, MAPK14 |
| Glyasperin C | MOL004811 | gancao | NOS2, ESR1, MAPK14 |
| Isotrifoliol | MOL004814 | gancao | NOS2, ESR1, MAPK14 |
| (E)-1-(2,4-dihydroxyphenyl)-3-(2,2-dimethylchromen-6-yl)prop-2-en-1-one | MOL004815 | gancao | NOS2, ESR1, MAPK14 |
| kanzonols W | MOL004820 | gancao | NOS2, ESR1, MAPK14 |
| (2S)-6-(2,4-dihydroxyphenyl)-2-(2-hydroxypropan-2-yl)-4-methoxy-2,3-dihydrofuro[3,2-g]chromen-7-one | MOL004824 | gancao | NOS2, ESR1, MAPK14 |
| Semilicoisoflavone B | MOL004827 | gancao | NOS2, ESR1 |
| Glepidotin A | MOL004828 | gancao | NOS2, ESR1, MAPK14 |
| Glepidotin B | MOL004829 | gancao | ESR1 |
| Phaseolinisoflavan | MOL004833 | gancao | NOS2, ESR1, MAPK14 |
| Glypallichalcone | MOL004835 | gancao | NOS2, ESR1, MAPK14 |
| 8-(6-hydroxy-2-benzofuranyl)-2,2-dimethyl-5-chromenol | MOL004838 | gancao | NOS2, ESR1 |
| Licochalcone B | MOL004841 | gancao | NOS2, ESR1, MAPK14 |
| licochalcone G | MOL004848 | gancao | NOS2, ESR1, MAPK14 |
| 3-(2,4-dihydroxyphenyl)-8-(1,1-dimethylprop-2-enyl)-7-hydroxy-5-methoxy-coumarin | MOL004849 | gancao | NOS2, ESR1, MAPK14 |
| Licoricone | MOL004855 | gancao | NOS2, ESR1 |
| Gancaonin A | MOL004856 | gancao | NOS2, ESR1 |
| Gancaonin B | MOL004857 | gancao | NOS2, ESR1 |
| 3-(3,4-dihydroxyphenyl)-5,7-dihydroxy-8-(3-methylbut-2-enyl)chromone | MOL004863 | gancao | NOS2, ESR1, MAPK14 |
| 5,7-dihydroxy-3-(4-methoxyphenyl)-8-(3-methylbut-2-enyl)chromone | MOL004864 | gancao | NOS2, ESR1, MAPK14 |
| Glycyrin | MOL004879 | gancao | NOS2, ESR1 |
| Licocoumarone | MOL004882 | gancao | ESR1 |
| Licoisoflavone | MOL004883 | gancao | NOS2, ESR1, MAPK14 |
| Licoisoflavone B | MOL004884 | gancao | NOS2, ESR1 |
| licoisoflavanone | MOL004885 | gancao | NOS2, ESR1 |
| shinpterocarpin | MOL004891 | gancao | NOS2, ESR1, OPRD1, OPRM1, MAPK14 |
| (E)-3-[3,4-dihydroxy-5-(3-methylbut-2-enyl)phenyl]-1-(2,4-dihydroxyphenyl)prop-2-en-1-one | MOL004898 | gancao | ESR1, MAPK14 |
| licopyranocoumarin | MOL004904 | gancao | NOS2, ESR1 |
| Glyzaglabrin | MOL004907 | gancao | NOS2, ESR1, MAPK14 |
| Glabridin | MOL004908 | gancao | NOS2, ESR1, MAPK14 |
| Glabranin | MOL004910 | gancao | NOS2, ESR1 |
| Glabrene | MOL004911 | gancao | NOS2, ESR1, MAPK14 |
| Glabrone | MOL004912 | gancao | NOS2, ESR1, MAPK14 |
| 1,3-dihydroxy-9-methoxy-6-benzofurano[3,2-c]chromenone | MOL004913 | gancao | ESR1, MAPK14 |
| 1,3-dihydroxy-8,9-dimethoxy-6-benzofurano[3,2-c]chromenone | MOL004914 | gancao | ESR1, MAPK14 |
| Eurycarpin A | MOL004915 | gancao | NOS2, ESR1, MAPK14 |
| Sigmoidin-B | MOL004935 | gancao | ESR1 |
| (2R)-7-hydroxy-2-(4-hydroxyphenyl)chroman-4-one | MOL004941 | gancao | ESR1 |
| (2S)-7-hydroxy-2-(4-hydroxyphenyl)-8-(3-methylbut-2-enyl)chroman-4-one | MOL004945 | gancao | NOS2, ESR1 |
| Isoglycyrol | MOL004948 | gancao | NOS2, ESR1 |
| Isolicoflavonol | MOL004949 | gancao | NOS2, ESR1 |
| HMO | MOL004957 | gancao | NOS2, ESR1, MAPK14 |
| 1-Methoxyphaseollidin | MOL004959 | gancao | NOS2, ESR1, MAPK14 |
| Quercetin der. | MOL004961 | gancao | NOS2, ESR1, MAPK14 |
| 3'-Hydroxy-4'-O-Methylglabridin | MOL004966 | gancao | NOS2, ESR1, MAPK14 |
| licochalcone a | MOL000497 | gancao | NOS2, ESR1, MAPK14, RELA, BCL2 |
| 3'-Methoxyglabridin | MOL004974 | gancao | NOS2, ESR1, MAPK14 |
| 2-[(3R)-8,8-dimethyl-3,4-dihydro-2H-pyrano[6,5-f]chromen-3-yl]-5-methoxyphenol | MOL004978 | gancao | NOS2, ESR1, MAPK14 |
| Inflacoumarin A | MOL004980 | gancao | ESR1 |
| Kanzonol F | MOL004988 | gancao | ESR1 |
| 6-prenylated eriodictyol | MOL004989 | gancao | NOS2, ESR1 |
| 7,2',4'-trihydroxy－5-methoxy-3－arylcoumarin | MOL004990 | gancao | NOS2, ESR1, MAPK14 |
| 7-Acetoxy-2-methylisoflavone | MOL004991 | gancao | NOS2, ESR1, MAPK14 |
| 8-prenylated eriodictyol | MOL004993 | gancao | ESR1 |
| Vestitol | MOL000500 | gancao | NOS2, ESR1, MAPK14 |
| Gancaonin G | MOL005000 | gancao | NOS2, ESR1, MAPK14 |
| Gancaonin H | MOL005001 | gancao | ESR1 |
| Licoagrocarpin | MOL005003 | gancao | NOS2, ESR1, MAPK14 |
| Glyasperins M | MOL005007 | gancao | NOS2, ESR1, |
| Glycyrrhiza flavonol A | MOL005008 | gancao | NOS2, ESR1, |
| Licoagroisoflavone | MOL005012 | gancao | NOS2, ESR1, MAPK14 |
| Odoratin | MOL005016 | gancao | NOS2, ESR1, MAPK14 |
| Phaseol | MOL005017 | gancao | ESR1, MAPK14, |
| Xambioona | MOL005018 | gancao | NOS2, ESR1 |
| dehydroglyasperins C | MOL005020 | gancao | NOS2, ESR1, MAPK14 |

Table S3: Signaling pathways of the KEGG enrichment analysis in the treatment of cough variant asthma with Zhisou San

| NO. | GO | Description | Count | Log10(P) | Hits |
| --- | --- | --- | --- | --- | --- |
| 1 | ko05142 | Chagas disease (American trypanosomiasis) | 15 | -29.2055 | AKT1, MAPK14, FOS, IFNG, IL1B, IL2, IL6, IL10, NOS2, MAPK3, MAPK8, MAPK10, RELA, CCL2, TNF |
| 2 | ko04657 | IL-17 signaling pathway | 13 | -24.8168 | AKT1, MAPK14, FOS, IFNG, IL1B, IL2, IL6, IL10, NOS2, MAPK3, MAPK8, MAPK10, RELA, CCL2, TNF |
| 3 | ko04668 | TNF signaling pathway | 13 | -23.9208 | MAPK14, FOS, IFNG, IL1B, IL4, IL6, CXCL10, MAPK3, MAPK8, MAPK10, RELA, CCL2, TNF |
| 4 | ko04933 | AGE-RAGE signaling pathway in diabetic complications | 12 | -22.0494 | MAPK14, FOS, IFNG, IL1B, IL4, IL6, CXCL10, MAPK3, MAPK8, MAPK10, RELA, CCL2, TNF |
| 5 | ko05145 | Toxoplasmosis | 12 | -21.3247 | AKT1, MAPK14, FOS, ICAM1, IL1B, IL6, CXCL10, MAPK3, MAPK8, MAPK10, RELA, CCL2, TNF |
| 6 | ko05164 | Influenza A | 13 | -21.1499 | AKT1, MAPK14, FOS, ICAM1, IL1B, IL6, CXCL10, MAPK3, MAPK8, MAPK10, RELA, CCL2, TNF |
| 7 | ko05133 | Pertussis | 11 | -21.059 | AKT1, BCL2, MAPK14, ICAM1, IL1B, IL6, MAPK3, MAPK8, MAPK10, RELA, CCL2, TNF |
| 8 | ko05152 | Tuberculosis | 13 | -20.9519 | AKT1, BCL2, MAPK14, ICAM1, IL1B, IL6, MAPK3, MAPK8, MAPK10, RELA, CCL2, TNF |
| 9 | ko05418 | Fluid shear stress and atherosclerosis | 12 | -20.0861 | AKT1, BCL2, BCL2L1, MAPK14, IFNG, IL10, NOS2, MAPK3, MAPK8, MAPK10, RELA, TNF |
| 10 | ko04620 | Toll-like receptor signaling pathway | 11 | -19.4757 | AKT1, BCL2, BCL2L1, MAPK14, IFNG, IL10, NOS2, MAPK3, MAPK8, MAPK10, RELA, TNF |
| 11 | hsa04625 | c-type lectin receptor signaling pathway | 11 | -19.3337 | AKT1, MAPK14, ICAM1, IFNG, IL1B, IL6, CXCL10, MAPK3, MAPK8, MAPK10, RELA, CCL2, TNF |
| 12 | ko04659 | Th17 cell differentiation | 11 | -19.3337 | MAPK14, FOS, IL1B, IL6, IL10, NOS2, MAPK3, MAPK8, MAPK10, RELA, TNF |
| 13 | ko05140 | Leishmaniasis | 10 | -18.8535 | AKT1, BCL2, MAPK14, IFNG, IL1B, IL6, IL10, NOS2, MAPK3, MAPK8, MAPK10, RELA, TNF |
| 14 | ko05132 | Salmonella infection | 10 | -18.1012 | AKT1, MAPK14, ICAM1, IFNG, IL1B, IL6, CXCL10, MAPK3, MAPK8, MAPK10, RELA, CCL2, TNF |
| 15 | hsa05169 | Epstein-Barr virus infection | 13 | -18.0041 | MAPK14, FOS, IL1B, IL6, IL10, NOS2, MAPK3, MAPK8, MAPK10, RELA, TNF |
| 16 | ko04660 | T cell receptor signaling pathway | 10 | -17.2821 | AKT1, BCL2, MAPK14, IFNG, IL1B, IL6, IL10, NOS2, MAPK3, MAPK8, MAPK10, RELA, TNF |
| 17 | ko04621 | NOD-like receptor signaling pathway | 11 | -17.0507 | AKT1, BCL2, MAPK14, FOS, ICAM1, IFNG, IL1B, MAPK8, MAPK10, RELA, CCL2, TNF |
| 18 | hsa05161 | Hepatitis B | 11 | -16.8539 | AKT1, BCL2, MAPK14, FOS, ICAM1, IFNG, IL1B, MAPK8, MAPK10, RELA, CCL2, TNF |
| 19 | ko04380 | Osteoclast differentiation | 10 | -16.2366 | AKT1, MAPK14, FOS, IL1B, IL6, CXCL10, MAPK3, MAPK8, MAPK10, RELA, TNF |
| 20 | ko04658 | Th1 and Th2 cell differentiation | 9 | -15.5662 | AKT1, MAPK14, IL1B, IL2, IL6, IL10, MAPK3, MAPK8, MAPK10, RELA, TNF |
| 21 | hsa05160 | Hepatitis C | 10 | -15.0713 | MAPK14, FOS, IFNG, IL1B, IL2, IL4, IL6, MAPK3, MAPK8, MAPK10, RELA |
| 22 | ko04071 | Sphingolipid signaling pathway | 9 | -14.5609 | AKT1, MAPK14, FOS, IL1B, IL6, CXCL10, MAPK3, MAPK8, MAPK10, RELA, TNF |
| 23 | hsa05167 | kaposi sarcoma-associated herpesvirus infection | 10 | -14.4634 | MAPK14, FOS, IFNG, IL1B, IL2, IL4, IL6, MAPK3, MAPK8, MAPK10, RELA |
| 24 | ko04917 | Prolactin signaling pathway | 8 | -14.398 | MAPK14, FOS, IFNG, IL1B, IL4, IL10, NOS2, MAPK3, RELA, TNF |
| 25 | hsa04010 | MAPK signaling pathway | 11 | -13.9994 | MAPK14, FOS, IFNG, IL1B, IL4, IL10, NOS2, MAPK3, RELA, TNF |
| 26 | ko04210 | Apoptosis | 9 | -13.9344 | MAPK14, FOS, IFNG, IL1B, IL6, NOS2, MAPK3, MAPK8, MAPK10, RELA |
| 27 | hsa05170 | human immunodeficiency virus 1 infection | 10 | -13.7366 | AKT1, BCL2, MAPK14, ICAM1, IFNG, IL6, IL10, CXCL10, MYC, MAPK8, MAPK10, RELA, TNF |
| 28 | hsa05163 | human cytomegalovirus infection | 10 | -13.5318 | MAPK14, FOS, IFNG, IL1B, IL6, NOS2, MAPK3, MAPK8, MAPK10, RELA |
| 29 | hsa01522 | Endocrine resistance | 8 | -13.258 | AKT1, MAPK14, FOS, IFNG, IL2, IL4, IL10, MAPK3, RELA, TNF |
| 30 | hsa05168 | Herpes simplex virus 1 infection | 12 | -12.8817 | BCL2, BCL2L1, MAPK14, IL1B, IL6, MAPK3, MAPK8, MAPK10, RELA, CCL2, TNF |
